# The Book World of Medicine and Science

**Published:** 1899-09-16

**Authors:** 


### The Book World of Medicine and Science.

Diseases of the Ear, Nose, and Throat, and their Accessory Cavities. By Seth Scott Bishop, M.D., D.C.L., LL.D. Second edition, thoroughly revised and enlarged. Illustrated with 94 coloured lithographs and 216 additional illustrations. (Philadelphia, New York, and Chicago: The F. A. Davis Company. 1898.)

The progress of knowledge demands new editions, and the book before us while giving a thoroughly good insight into the present position of medicine and surgery in regard to the subjects on which it treats, serves also to show how considerable have been the advances which have taken place in them during the last few years. The book is primarily the book of a specialist, and without going so far as to say that it is one for a specialist, its scope, and especially the earlier part of the volume which deals with instruments and appliances, tend to impress the reader strongly with the idea that if all the beautiful arrangements depicted are necessary for the practice of otology, rhinology, and laryngology, the ordinary general practitioner must humbly take his place outside the charmed circle. Notwithstanding, however, the somewhat ostentatious display of apparatus and flourish of instruments which decorate its opening pages, the book is one of very great value and is full of information. The descriptions are accurate, the treatment described and recommended is in most cases quite up to date and such as may be trusted to prove useful, and the anatomical descriptions scattered throughout the pages, which are profusely and most graphically illustrated, serve to make it easy to follow the text. So far as saving of time is concerned, it is an immense advantage to have anatomical and such like matters described and illustrated on the spot, just where they arise, instead of being referred to other works which may not at the moment happen to be at hand. The work, which is evidently the outcome of much care, labour, and experience, may properly be accepted as a good handbook of the subjects which are treated in its pages.

A Text Book of Practical Obstetrics. By Egbert H. Grandin, M.D., with the collaboration of George W. Jarman, M.D. Second edition, revised and enlarged. Illustrated with 64 full-page photographic plates and 86 illustrations in the text. (Philadelphia, New York, and Chicago: The F. A. Davis Company. 1898. Price 4 dols.)

In many ways this is an attractive book. In some ways, and to some people, perhaps, it will prove repulsive. Its attractiveness is this, that it deals with obstetrics as a mere scientific problem. Given a woman with a child in her womb, the problem is how to get it out with the greatest safety, and how to get it out alive if that be possible. Of course that is always the problem, but it is too apt in some hands to be interfered with by the idea that parturition is but a normal process, and that although complicated labour is to be conducted on the lines of a surgical operation, normal labour is not to be interfered with, but may be left to a midwife or be allowed to take place smothered up in the bed clothes and out of sight. The attempt to draw a definite line between normal and abnormal labour is the origin of many obstetric sins. While, however, the authors of the book before us would apparently deal with every case from the beginning with full preparedness for all eventualities, and thus according to the ideas of some people would make a great to-do about an ordinary physiological process, it is to be noted that their definition of normal labour is wide, and such as to include cases which, according to many, should be looked upon as distinctly abnormal. "Under the term 'normal labour' are understood instances where the fœtus enters the pelvic inlet and emerges at the pelvic outlet after a fashion in accordance with the normal mechanism of labour. Engagement, descent, rotation, extension, and delivery occur after that fashion which is essential for the welfare of the woman and the child, under the natural efforts, and within that time-limit which, according to the individual case, is consistent with the conservation of the strength of the woman and the vitality of the fœtus. Under this definition any variety of presentation may be normal, since, as we have noted, presentations of the pelvic extremity and of the face may be terminated within the stated limits and with the stated results."

When we say that to some the book will appear repulsive we refer more especially to the illustrations, a series of large-size reproductions of what are apparently photographic "snap shots" of the actual details of the proceedings referred to in the text, *i.e.*, steps in the conduct of labour. But the Americans are a practical people, and this book is meant to teach; and there can be but little doubt that, given even the best written description in the world, such description will approach still nearer in vividness and actuality to what is to be obtained from clinical lectures and bedside demonstrations, if it is illustrated by well-chosen photographs of the proceedings described. In this book the dorsal position is the one recommended, and the chapter on normal labour is illustrated by a series of photographs of its every stage, from the preliminary catheterisation to the final expression of the placenta and the application of the binder. By some people all this rush to photography and this pictorial illustration of things that most books are content to describe in words is likely to be considered an abuse of the facilities offered by the camera. But there is no doubt about the realism of it all, and we can have but little doubt about the effectiveness of such pictures from a teaching point of view. Still it is not a book to leave about. Take it altogether, this work is one which ought to give the student a good working knowledge of midwifery, especially if he has the opportunity of practising upon the model the operative proceedings which are described. There can be but little doubt that every student ought to take far more pains than is usually done to render himself practically familiar with the use and with the "feel" of every obstetric instrument, by frequent practice on the model, before he undertakes operative midwifery. The teachings of the book in regard to asepsis will be found fully up to, and, in fact, we should say rather beyond what may be taken as the modern standpoint. We are told that "before undertaking any surgical manipulation the following means should be resorted to: The external genitals are to be scrubbed with hot soap and water, and next washed with a solution of bichloride (1 to 1,000). If the required manipulations are in the vagina a new tooth-brush should be inserted into the canal, and this should also be scrubbed with a solution of mercury (1 to 1,000)." Of course, something depends upon what is meant by "surgical manipulations."

